# Are we Using the Right Evidence to Inform Suicide Prevention in Low- and Middle-Income Countries? An Umbrella Review

**DOI:** 10.1080/13811118.2024.2322144

**Published:** 2024-03-13

**Authors:** Ivie Itua, Kamal Shah, Patrick Galway, Faiza Chaudhry, Tatiana Georgiadi, Juhi Rastogi, Shameer Naleer, Duleeka Knipe

**Keywords:** Global mental health, self-harm, suicide

## Abstract

**Objective:**

Suicide disproportionately affects low- and middle-income countries and evidence regarding prevention approaches developed in high income countries may not be applicable in these settings. We conducted an umbrella review to assess whether the conclusions of suicide prevention systematic reviews accurately reflect the studies contained within those reviews in terms of setting generalizability.

**Methods:**

We conducted database searches in PubMed/Medline, Embase, PsycInfo, PsychExtra, OVID global health, and LILACS/BECS. We included systematic reviews with the outcome of suicide, including bereavement studies where suicide death was also the exposure.

**Results:**

Out of the 147 reviews assessed, we found that over 80% of systematic reviews on suicide deaths do not provide an accurate summary of review findings with relation to geographic relevance and ultimately generalizability.

**Conclusion:**

Systematic reviews are often the resource used by practitioners and policymakers to guide services. Misleading reviews can detrimentally impact suicide prevention efforts in LMICs. We call for systematic reviewers to be responsible when generalizing the findings of their reviews particularly in the abstracts.

Suicide is a major global issue with 800,000 people dying every year − 77% of these deaths occurring in low- and middle-income countries (LMICs) (World Health Organisation, 2021). We know relatively little about this behavior in these contexts. A recent review has shown that whilst there has been a steady increase in the number of academic publications in suicide research, there are few papers originating from LMICs (Cai et al., 2020).

Suicidal behavior is complex. No single factor will increase the risk of suicide, rather it is the complex interplay of multiple factors. Given the significant variability in cultural, economic, and medical care between countries, the findings in one might not be applicable to another. This is particularly likely to be the case for research that is purely conducted in high-income countries (HIC). Despite the sparse evidence base regarding suicide in LMICs, the available evidence in terms of both the epidemiology and risk factors suggests that suicidal behavior in these contexts is likely to be different to that of HIC (Knipe et al., 2022).

With the growing body of research evidence, policy makers and practitioners often turn to systematic reviews to provide them with a summary of the most up to date evidence. Against the backdrop of suicide research primarily originating from HIC, it would be reasonable to assume that the findings from these systematic reviews would only be applicable to high-income settings. However, often these reviews will make recommendations without the caveat that the results of the review are only applicable to a high-income setting (Knipe & Jewkes, 2022). Ultimately these important reviews might be quite misleading.

We conducted an umbrella review of systematic reviews to assess whether the current systematic review evidence base informing suicide prevention globally provides an accurate summary of the studies contained within those reviews.

## METHODS

We registered the umbrella review protocol with PROSPERO (registration ID: CRD42020198194). Umbrella reviews are essentially a systematic review of systematic reviews.

### Search Strategy

We conducted database searches in PubMed/Medline, Embase, PsycInfo, PsychExtra, OVID global health, and LILACS/BECS. We used the search terms suicide (suicid*) AND review. No date restrictions were applied and searches were conducted on 23 July 2020. The full text versions of studies that potentially fit the inclusion criteria were reviewed by two independent screeners after the titles and abstracts of the articles were screened for relevance (two independent reviewers).

### Inclusion and Exclusion Criteria

Inclusion and exclusion criteria are summarized in Table 1. We included studies with the outcome of suicide, including bereavement studies where suicide death was also the exposure. We included studies which were systematic reviews, and excluded all other reviews (e.g., scoping reviews). We also excluded records related to conference abstracts and protocols where the research had not been completed and/or published. Due to the vast nature of this topic, we focussed on reviews on death by suicide and excluded reviews that studied other suicidal behavior, intent, and ideation. Some reviews looked at multiple related outcomes including death by suicide but did not distinguish between the outcomes and thus were excluded. We excluded reviews that did not look at death by suicide as a main outcome, i.e., reviews that have less than five original studies examining death by suicide. We excluded reviews that examined medically assisted suicide, and those focused on clinical or specific subgroups of the population because the findings might not be representative of the general population. Although we excluded studies that examine suicide of people living with mental health conditions, some reviews examined suicide and mental health conditions as separate entities and thus were included. Where the full text of the article couldn’t be found, the authors were contacted to provide the full text where possible. Full text articles that were in different languages, were analyzed after being translated to English using Google translate. When this was not possible, at least 3 attempts were made to contact authors to provide translation and/or further information.

**TABLE 1.** Inclusion and exclusion criteria.

Inclusion criteria	Exclusion criteria
All ages and genders	Broader mental health interventions
All populations in all countries	Studies that examine attitudes toward
Reviews studying general population	Examining suicidal ideation or self-injury or suicide attempts
Death by suicide	Other types of reviews (e.g. scoping reviews)
Systematic reviews or meta-analyses	Studies specific population that does not represent general population
English text/translations available	Medically assisted suicide
Peer reviewed journal	

Unlike typical umbrella reviews which are focused on summarizing the strength of the evidence of for example a particular exposure to a condition, or an intervention effect, our umbrella review’s objective focused on how systematic review authors present their findings. For this reason, we included all eligible systematic reviews even if the underlying studies within the reviews overlap.

### Data Extraction

We extracted data in a pre-piloted form and classified the included studies by income using the 2020 World Bank classification (World Bank, n.d.). When a review examined suicide death as an exposure and outcome, we extracted data for both (Hill et al., 2020).

All papers were screened, and data extracted, by two independent reviewers. Discrepancies were resolved through team meetings or decided by the senior author (DK). We did not formally assess risk of bias of the included systematic reviews. Risk of bias in umbrella reviews is designed to assess whether the review has been designed, conducted, analyzed and/or reported in such a way as to introduce systemic deviations from the truth (Pollock M et al., 2023). The reason to do this is so that there is a way of assessing whether the strength of the evidence is likely to be impacted by bias—i.e., internal validity. As our objective was not to assess the strength of the association, but to assess the quality of the review in terms of the recommendations that are put forward (i.e., external validity), we decided that a risk of bias assessment would not be needed. The results of a risk of bias assessment would not have been used to either present or interpret the findings of this review.

### Analysis

We calculated the proportion of included studies in each review that were based in HICs, low income, lower-middle income, and upper-middle income countries. We then evaluated how the conclusions (as presented in the abstract and discussion) of each review accurately reflect the generalizability of the review in terms of country/income data. We did not conduct a formal statistical synthesis as this would have not been appropriate for the objectives of this study.

## RESULTS

**FIGURE 1.**
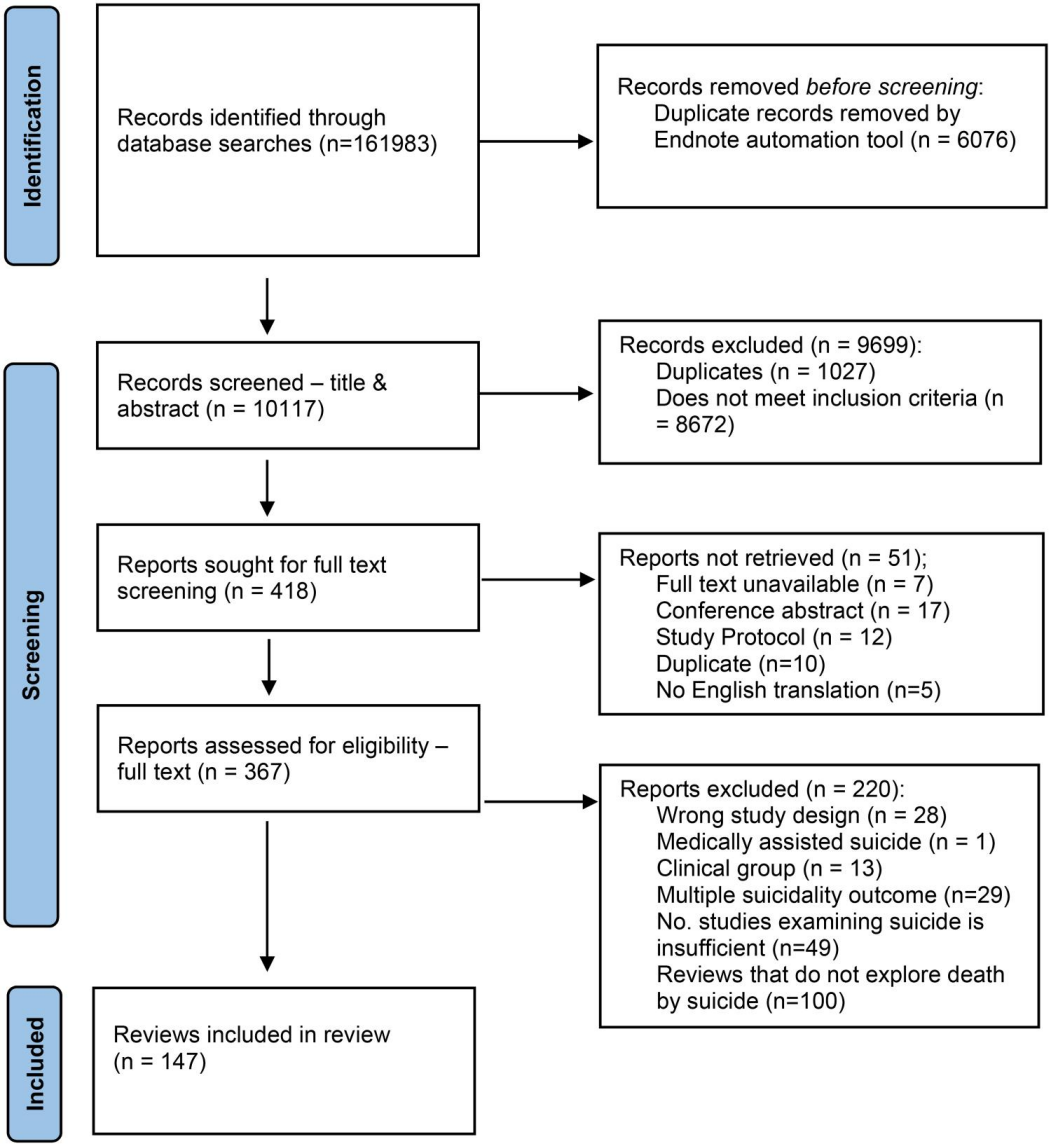
PRISMA Flow diagram of included studies.

The electronic searches generated 10117 de-duplicated records, with 147 systematic reviews meeting the inclusion criteria (Figure 1).

Less than 1 in 5 of the systematic reviews were led (i.e., first author) by researchers in middle-income countries and none were led by researchers in low-income countries. The details of included studies can be found in Table 2. There were 4595 included studies combined from 147 reviews, but only 2758 of those studies investigated suicide deaths. Of the 2758 studies included within the reviews, 68% were conducted in HICs, 8% from upper middle-income countries, 6% from low middle income countries and only 1 study was from a low-income country. 30 of the studies were from a mixture of countries and the country was unknown for 17% of the 2758 included studies.

**TABLE 2.** Characteristics of included studies*.

Review	No. of primary studies included that examine suicide death	No. of studies by World Bank Income Status	Does the review conclusions reflect the generalizability of the study?
Ahmed et al. (2017)	8	HIC:0 UMIC:0 LMIC:8 LIC:0 NK:0	Yes
Aho et al. (2017)	12	HIC:0 UMIC:0 LMIC:0 LIC:0 NK:12	?
Amiri and Behnezhad (2018)	9	HIC:9 UMIC:0 LMIC:0 LIC:0 NK:0	No
Amiri and Behnezhad (2020)	17	HIC:17 UMIC:0 LMIC:0 LIC:0 NK:0	No
Andriessen et al. (2016)	56	HIC:54 UMIC:1 LMIC:1 LIC:0 NK:0	No
Andriessen et al. (2018)	11	HIC:10 UMIC:0 LMIC:0 LIC:1 NK:0	No
Andriessen, Krysinska, Hill, et al. (2019)	12	HIC:12 UMIC:0 LMIC:0 LIC:0 NK:0	No
Andriessen, Krysinska, Kolves, et al. (2019)	8	HIC:8 UMIC:0 LMIC:0 LIC:0 NK:0	No
Anglemyer et al. (2014)	14	HIC:14 UMIC:0 LMIC:0 LIC:0 NK:0	No
Anguelova et al. (2003)	11	HIC:11 UMIC:0 LMIC:0 LIC:0 NK:0	No
Antypa et al. (2013)	20	HIC:15 UMIC:4 LMIC:1 LIC:0 NK:0	Yes
Arsenault-Lapierre et al. (2004)	27	HIC:25 UMIC:1 LMIC:1 LIC:0 NK:0	No
Barjasteh-Askari et al. (2020)	14	HIC:14 UMIC:0 LMIC:0 LIC:0 NK:0	No
Barker et al. (2017)	9	HIC:9 UMIC:0 LMIC:0 LIC:0 NK:0	No
Barry et al. (2020)	36	HIC:36 UMIC:0 LMIC:0 LIC:0 NK:0	No
Belsher et al. (2019)	11	HIC:10 UMIC:0 LMIC:1 LIC:0 NK:0	No
Ben Alaya et al. (2018)	6	HIC:0 UMIC:0 LMIC:6 LIC:0 NK:0	Yes
Blacker et al. (2019)	12	HIC:10 UMIC:1 LMIC:1 LIC:0 NK:0	Yes
Brezo et al. (2006)	11	HIC:0 UMIC:0 LMIC:0 LIC:0 NK:11	?
Brown et al. (2017)	8	HIC:2 UMIC:5 LMIC:0 LIC:0 NK:0 M:1	Yes
Cairns et al. (2017)	20	HIC:19 UMIC:0 LMIC:0 LIC:0 NK:0 M:1	Yes
Calati et al. (2015)	8	HIC:4 UMIC:0 LMIC:1 LIC:0 NK:3	No
Cano-Montalbán and Quevedo-Blasco (2018)	30	HIC:20 UMIC:9 LMIC:0 LIC:0 NK:0 M:1	No
Causer et al. (2019)	12	HIC:10 UMIC:2 LMIC:0 LIC:0 NK:0	No
Cavanagh et al. (2003)	76	HIC:0 UMIC:0 LMIC:0 LIC:0 NK:76	?
Cenderadewi et al. (2020)	10	HIC:10 UMIC:0 LMIC:0 LIC:0 NK:0	Yes
Cho et al. (2016)	48	HIC:36 UMIC:7 LMIC:5 LIC:0 NK:0	Yes
Colucci and Martin (2007)	10	HIC:5 UMIC:1 LMIC:0 LIC:0 NK:0 M:4	No
Cordner et al. (2020)	31	HIC:25 UMIC:5 LMIC:0 LIC:0 NK:1	No
Cox et al. (2013)	14	HIC:14 UMIC:0 LMIC:0 LIC:0 NK:0	No
De la Cruz-Cano (2017)	7	HIC:7 UMIC:0 LMIC:0 LIC:0 NK:0	No
de Souza et al. (2020)	9	HIC:0 UMIC:9 LMIC:0 LIC:0 NK:0	Yes
Dickson et al. (2019)	11	HIC:11 UMIC:0 LMIC:0 LIC:0 NK:0	Yes
Dong et al. (2015)	20	HIC:0 UMIC:0 LMIC:0 LIC:0 NK:20	Yes
Duarte et al. (2020)	9	HIC:8 UMIC:1 LMIC:0 LIC:0 NK:0	No
Dutheil et al. (2019)	52	HIC:0 UMIC:0 LMIC:0 LIC:0 NK:52	?
El-Sayed et al. (2012)	12	HIC:12 UMIC:0 LMIC:0 LIC:0 NK:0	No
Freire and Koifman (2013)	14	HIC:9 UMIC:5 LMIC:0 LIC:0 NK:0	No
Fung and Chan (2011)	17	HIC:16 UMIC:1 LMIC:0 LIC:0 NK:0	No
Galvão et al. (2018)	50	HIC:38 UMIC:9 LMIC:1 LIC:0 NK:0 M:2	Yes
Gao et al. (2019)	16	HIC:12 UMIC:4 LMIC:0 LIC:0 NK:0	Yes
Glenn et al. (2020)	45	HIC:37 UMIC:6 LMIC:2 LIC:0 NK:0	Yes
González-Castro et al. (2017)	5	HIC:5 UMIC:0 LMIC:0 LIC:0 NK:0	No
Goodday et al. (2019)	5	HIC:5 UMIC:0 LMIC:0 LIC:0 NK:0	No
Gorton et al. (2016)	11	HIC:11 UMIC:0 LMIC:0 LIC:0 NK:0	No
Gramaglia et al. (2019)	6	HIC:6 UMIC:0 LMIC:0 LIC:0 NK:0	No
Grandclerc et al. (2016)	5	HIC:5 UMIC:0 LMIC:0 LIC:0 NK:0	No
Grek (2007)	26	HIC:0 UMIC:0 LMIC:0 LIC:0 NK:26	?
Gunnell et al. (2005)	15	HIC:14 UMIC:0 LMIC:1 LIC:0 NK:0	No
Gunnell et al. (2017)	27	HIC:18 UMIC:2 LMIC:7 LIC:0 NK:0	Yes
Hahn et al. (2014)	7	HIC:7 UMIC:0 LMIC:0 LIC:0 NK:0	No
Havârneanu et al. (2015)	9	HIC:9 UMIC:0 LMIC:0 LIC:0 NK:0	No
Haw and Hawton (2016)	20	HIC:19 UMIC:0 LMIC:1 LIC:0 NK:0	Yes
Hill et al. (2020)	27	HIC:20 UMIC:4 LMIC:3 LIC:0 NK:0	No
Hofstra et al. (2020)	9	HIC:7 UMIC:0 LMIC:2 LIC:0 NK:0	No
Holmes et al. (2012)	10	HIC:2 UMIC:8 LMIC:0 LIC:0 NK:0	No
Honkaniemi et al. (2017)	16	HIC:16 UMIC:0 LMIC:0 LIC:0 NK:0	Yes
Hua et al. (2019)	26	HIC:21 UMIC:1 LMIC:0 LIC:0 NK:2 M:2	No
Hunt et al. (2017)	6	HIC:6 UMIC:0 LMIC:0 LIC:0 NK:0	No
Ide et al. (2010)	8	HIC:8 UMIC:0 LMIC:0 LIC:0 NK:0	No
Iemmi et al. (2016)	17	HIC:1 UMIC:6 LMIC:9 LIC:0 NK:0 M:1	Yes
Jafari et al. (2020)	12	HIC:10 UMIC:1 LMIC:1 LIC:0 NK:0	No
Jang and Elfenbein (2019)	5	HIC:0 UMIC:0 LMIC:0 LIC:0 NK:5	?
Kenedi et al. (2016)	11	HIC:0 UMIC:0 LMIC:0 LIC:0 NK:11	?
Kleck (2019)	29	HIC:29 UMIC:0 LMIC:0 LIC:0 NK:0	No
Klingelschmidt et al. (2018)	32	HIC:32 UMIC:0 LMIC:0 LIC:0 NK:0	No
Klinitzke et al. (2013)	12	HIC:11 UMIC:0 LMIC:0 LIC:0 NK:0 M:1	No
Knipe et al. (2019)	22	HIC:0 UMIC:13 LMIC:9 LIC:0 NK:0	Yes
Knipe et al. (2015)	5	HIC:0 UMIC:1 LMIC:4 LIC:0 NK:0	Yes
Kõlves et al. (2013)	19	HIC:11 UMIC:7 LMIC:1 LIC:0 NK:0	No
Kuramoto et al. (2009)	9	HIC:9 UMIC:0 LMIC:0 LIC:0 NK:0	No
Large et al. (2009)	49	HIC:48 UMIC:1 LMIC:0 LIC:0 NK:0	Yes
Li and Katikireddi (2019)	24	HIC:0 UMIC:24 LMIC:0 LIC:0 NK:0	Yes
Li et al. (2012)	5	HIC:0 UMIC:5 LMIC:0 LIC:0 NK:0	Yes
Linde et al. (2017)	7	HIC:0 UMIC:0 LMIC:0 LIC:0 NK:7	?
Lindeman et al. (1996)	14	HIC:13 UMIC:1 LMIC:0 LIC:0 NK:0	No
Liotta et al. (2015)	9	HIC:7 UMIC:1 LMIC:1 LIC:0 NK:0	No
Liu et al. (2021)	10	HIC:8 UMIC:2 LMIC:0 LIC:0 NK:0	No
Liu and Miller (2014)	20	HIC:10 UMIC:4 LMIC:5 LIC:0 NK:1	No
Malakouti et al. (2015)	12	HIC:2 UMIC:1 LMIC:9 LIC:0 NK:0	Yes
Maple et al. (2017)	27	HIC:21 UMIC:2 LMIC:4 LIC:0 NK:0	No
McDaid et al. (2008)	8	HIC:0 UMIC:0 LMIC:0 LIC:0 NK:8	?
Memon et al. (2020)	15	HIC:15 UMIC:0 LMIC:0 LIC:0 NK:0	No
Mew et al. (2017)	6	HIC:0 UMIC:0 LMIC:0 LIC:0 NK:6	?
Milner, Page, et al. (2013)	10	HIC:9 UMIC:0 LMIC:0 LIC:0 NK:0 M:1	No
Milner et al. (2014)	5	HIC:5 UMIC:0 LMIC:0 LIC:0 NK:0	No
Milner, Spittal, et al. (2013)	34	HIC:34 UMIC:0 LMIC:0 LIC:0 NK:0	No
Milner, Sveticic, et al. (2013)	29	HIC:17 UMIC:9 LMIC:3 LIC:0 NK:0	Yes
Milner et al. (2018)	6	HIC:6 UMIC:0 LMIC:0 LIC:0 NK:0	No
Milner et al. (2017)	9	HIC:8 UMIC:0 LMIC:1 LIC:0 NK:0	No
Milner et al. (2015)	5	HIC:5 UMIC:0 LMIC:0 LIC:0 NK:0	No
Miranda-Mendizabal et al. (2019)	8	HIC:7 UMIC:1 LMIC:0 LIC:0 NK:0	No
Mishara and Bardon (2016)	55	HIC:19 UMIC:1 LMIC:0 LIC:0 NK:35	?
Morovatdar et al. (2013)	19	HIC:3 UMIC:2 LMIC:14 LIC:0 NK:0	Yes
Nazarzadeh et al. (2013)	7	HIC:0 UMIC:0 LMIC:7 LIC:0 NK:0	Yes
Niederkrotenthaler et al. (2020)	31	HIC:31 UMIC:0 LMIC:0 LIC:0 NK:0	No
Niedzwiedz et al. (2014)	82	HIC:68 UMIC:2 LMIC:1 LIC:0 NK:4 M:7	?
Okolie et al. (2017)	11	HIC:11 UMIC:0 LMIC:0 LIC:0 NK:0	No
Okolie et al. (2020)	14	HIC:14 UMIC:0 LMIC:0 LIC:0 NK:0	No
Owens et al. (2002)	26	HIC:26 UMIC:0 LMIC:0 LIC:0 NK:0	No
Oyama et al. (2008)	5	HIC:5 UMIC:0 LMIC:0 LIC:0 NK:0	Yes
Oyesanya et al. (2015)	38	HIC:19 UMIC:2 LMIC:0 LIC:0 NK:13 M:4	?
Panczak et al. (2013)	27	HIC:26 UMIC:1 LMIC:0 LIC:0 NK:0	Yes
Perera et al. (2016)	21	HIC:20 UMIC:0 LMIC:0 LIC:0 NK:0 M:1	No
Pigeon et al. (2016)	5	HIC:2 UMIC:1 LMIC:0 LIC:0 NK:2	No
Pirkis and Burgess (1998)	24	HIC:24 UMIC:0 LMIC:0 LIC:0 NK:0	No
Pirkis et al. (2015)	18	HIC:18 UMIC:0 LMIC:0 LIC:0 NK:0	No
Platt et al. (2010)	19	HIC:19 UMIC:0 LMIC:0 LIC:0 NK:0	Yes
Plöderl and Tremblay (2015)	7	HIC:2 UMIC:0 LMIC:0 LIC:0 NK:5	No
Pollock et al. (2018)	99	HIC:70 UMIC:17 LMIC:12 LIC:0 NK:0	Yes
Ragguett et al. (2017)	11	HIC:10 UMIC:1 LMIC:0 LIC:0 NK:0	No
Rane and Nadkarni (2014)	36	HIC:0 UMIC:0 LMIC:36 LIC:0 NK:0	Yes
Rehkopf and Buka (2006)	86	HIC:0 UMIC:0 LMIC:0 LIC:0 NK:86	?
Rice and Sher (2016)	?	HIC:? UMIC:? LMIC:? LIC:? NK:?	?
Robinson et al. (2018)	12	HIC:10 UMIC:0 LMIC:2 LIC:0 NK:0	No
Rowell et al. (2008)	9	HIC:9 UMIC:0 LMIC:0 LIC:0 NK:0	Yes
Runeson et al. (2017)	10	HIC:0 UMIC:0 LMIC:0 LIC:0 NK:10	?
Sales et al. (2019)	14	HIC:6 UMIC:0 LMIC:0 LIC:0 NK:8	No
Sargeant et al. (2018)	8	HIC:8 UMIC:0 LMIC:0 LIC:0 NK:0	Yes
Serafini et al. (2013)	6	HIC:0 UMIC:0 LMIC:0 LIC:0 NK:6	?
Shahid and Hyder (2008)	9	HIC:0 UMIC:0 LMIC:9 LIC:0 NK:0	Yes
Shields et al. (2017)	11	HIC:11 UMIC:0 LMIC:0 LIC:0 NK:0	No
Silva et al. (2014)	12	HIC:0 UMIC:0 LMIC:0 LIC:0 NK:12	?
Simon et al. (2013)	35	HIC:13 UMIC:22 LMIC:0 LIC:0 NK:0	Yes
Sisask and Värnik (2012)	48	HIC:48 UMIC:0 LMIC:0 LIC:0 NK:0	No
Skinner and Farrington (2020)	15	HIC:15 UMIC:0 LMIC:0 LIC:0 NK:0	No
Soole et al. (2015)	15	HIC:14 UMIC:0 LMIC:0 LIC:0 NK:0 M:2	No
Spallek et al. (2015)	24	HIC:24 UMIC:0 LMIC:0 LIC:0 NK:0	Yes
Spillane et al. (2017)	27	HIC:26 UMIC:1 LMIC:0 LIC:0 NK:0	No
Stanley et al. (2016)	43	HIC:39 UMIC:0 LMIC:0 LIC:0 NK:3 M:1	No
Stene-Larsen and Reneflot (2019)	44	HIC:44 UMIC:0 LMIC:0 LIC:0 NK:0	No
Sveen and Walby (2008)	41	HIC:0 UMIC:0 LMIC:0 LIC:0 NK:41	?
Szücs et al. (2018)	12	HIC:12 UMIC:0 LMIC:0 LIC:0 NK:0	No
Thompson et al. (2018)	17	HIC:13 UMIC:4 LMIC:0 LIC:0 NK:0	No
Tøllefsen et al. (2012)	31	HIC:30 UMIC:0 LMIC:1 LIC:0 NK:0	Yes
Too et al. (2014)	11	HIC:11 UMIC:0 LMIC:0 LIC:0 NK:0	No
Torok et al. (2017)	7	HIC:7 UMIC:0 LMIC:0 LIC:0 NK:0	No
Troya et al. (2019)	16	HIC:14 UMIC:0 LMIC:1 LIC:0 NK:0 M:1	No
Wagner et al. (2021)	6	HIC:6 UMIC:0 LMIC:0 LIC:0 NK:0	No
Walby et al. (2018)	35	HIC:35 UMIC:0 LMIC:0 LIC:0 NK:0	Yes
Wilson et al. (2020)	7	HIC:0 UMIC:0 LMIC:0 LIC:0 NK:7	?
Witt et al. (2019)	11	HIC:11 UMIC:0 LMIC:0 LIC:0 NK:0	No
Witt et al. (2017)	7	HIC:5 UMIC:2 LMIC:0 LIC:0 NK:0	No
Wu et al. (2016)	6	HIC:0 UMIC:0 LMIC:0 LIC:0 NK:6	?
Yoshimasu et al. (2008)	24	HIC:20 UMIC:2 LMIC:2 LIC:0 NK:0	No
Zhang et al. (2013)	10	HIC:9 UMIC:0 LMIC:0 LIC:0 NK:0 M:1	Yes
Zhao et al. (2018)	5	HIC:5 UMIC:0 LMIC:0 LIC:0 NK:0	Yes

^*^ HIC: High income country; UMIC: Upper middle income country; LMIC: Lower middle income country; LIC: Lower income country; NK: Country income status not reported/known; M: Multicounty data presented and income status unable to be assessed. ; ? : indicates where the study has not provided sufficient details regarding the income of the countries included in their review to assess whether the conclusions are supported by the included studies.

One review did not have any data on included studies (i.e., did not provide information on included studies) and thus was unable to be analyzed (Rice & Sher, 2016).

Of all the reviews, 17% (25/147) were focused on a specific region of the world (e.g., LMIC), whereas most reviews aimed to review the global literature. Of the 122 reviews that did not focus on a specific region, the median number of papers included in these reviews that were from LMIC were 0 (range: 0–17). Of note, we were unable to assess whether 21 of the reviews included data that would make the conclusions appropriate (i.e., generalizable) because the authors did not provide sufficient country data for all included studies). Despite the weight of evidence in favor of HICs, the majority of reviewers presented the conclusions of their review as if the evidence base was generalizable to a global population (83/101 (82%)).

## DISCUSSION

The findings of this umbrella review highlight that over 80% of systematic reviews on suicide deaths do not provide an accurate summary of review findings with relation to geographic relevance and ultimately generalizability. The reviews had conclusions and abstracts that made generalizations to the global population, whereas their included studies did not reflect a global population and were instead mostly reflective of HICs with less than 10% of the included studies in the reviews being from LMICs.

The findings of this review of reviews is in keeping with normative, although inappropriate, practices in academic publishing, where findings from HICs are reported as if they are applicable to all (Knipe & Jewkes, 2022). Systematic review authors in the field of suicide prevention are misrepresenting the findings of their reviews, which ultimately will misguide the users of their research. Our review has shown that 4-in-5 systematic reviews informing suicide prevention globally does not provide an accurate summary of the studies contained within those reviews. Whether systematic review authors believe that their work can indeed be generalized, or whether this is even a consideration is unknown. The majority of systematic reviews included in this review of reviews were led by researchers in HICs and therefore are more likely to see the studies included in their studies as “normal”—they might fail to see the absence of data from LMICs. However, the extrapolation of findings from a select number of “WEIRD” (Western, educated, industrialized, rich, democratic) countries to the rest of the world is likely to be inappropriate in the context of suicide prevention. The contextual differences between HICs and LMICs, and also within the vast grouping of LMICs needs to be appropriately recognized. Considering other contexts, cultures, socioeconomic settings should be kept in mind throughout the research process, when data are being presented and when it is being published.

If research has been done in a particular setting, i.e., HICs, then that should be reflected in the conclusions of the reviews and made clear in the abstracts. The importance of generalizability has been shown in research where interventions researched in one setting has been applied to another setting and not had the desired effect (in this instance the reduction of repeat self-harm behavior (Husain et al., 2014)). Critical evaluations of the evidence’s local applicability prior to adapting it to a different health setting are essential to maximizing its utility. Generalizability being highlighted in systematic reviews that are used to inform changes would help in making these evaluations. More scrutinization should be done during the drafting of manuscripts as well as in the peer-review and publishing process to avoid the use of irrelevant research in policy making (Knipe & Jewkes, 2022).

## STRENGTHS AND LIMITATIONS

This is the first review of reviews to consider the importance of context in the presentation of review findings. We employed robust systematic review processes; however, the findings of this review need to be considered in light of its limitations. First, although effort was made to search a variety of search engines, the ones that were used may not index as many non-English journals. Thus, we may have not identified important systematic reviews especially from LMICs where English may not the predominant language. However, the search engines used are some of the most used for systematic reviews. One can argue that a paper that cannot be easily found is less likely to be used to inform policy thus the systematic reviews presented in this review are of high importance when considering suicide prevention policy making. Secondly, one of the included systematic reviews investigated suicide in LMICs however it included studies from countries that are high income and did not include any studies from LICs. It is possible that the income status has changed from when the systematic review was completed to when this current systematic review is being completed. This highlights a possible limitation of this study as it is assessing research based on current income status and not previously. However, this review is being used to highlights the need for more research to be conducted in current LMICs and for researchers to be more aware of the way they present the generalizability of their research. This point still stands with the current review outcome.

## CONCLUSION

The findings of research in one context should not be assumed to be applicable to another setting. Countries which have similar characteristics and systems may share commonalities in terms of suicide prevention, but when these characteristics are vastly different, as is the case between many HICs and LMICs, caution needs to be employed in the presentation of result. As systematic reviews are key sources of information for policy makers, it is crucial that these reviews are representing the data within them correctly. Inadequate representation may lead to economically resource poor countries investing in prevention approaches which might be ineffective. The long-term consequences of this might lead to despondency by policy makers and the public, which is likely to be detrimental for suicide prevention. To avoid this there needs to be a concerted effort by researchers, peer-reviewers, and editors to ensure findings are accurately reported.
